# Opportunities for Overcoming *Mycobacterium tuberculosis* Drug Resistance: Emerging Mycobacterial Targets and Host-Directed Therapy

**DOI:** 10.3390/ijms20122868

**Published:** 2019-06-12

**Authors:** Eveline Torfs, Tatiana Piller, Paul Cos, Davie Cappoen

**Affiliations:** Laboratory of Microbiology, Parasitology and Hygiene (LMPH), Department of Pharmaceutical Sciences, University of Antwerp, Universiteitsplein 1, 2610 Wilrijk (Antwerpen), Belgium; eveline.torfs@uantwerpen.be (E.T.); tatiana.piller@uantwerpen.be (T.P.); paul.cos@uantwerpen.be (P.C.)

**Keywords:** *Mycobacterium tuberculosis*, drug resistance, antimycobacterial, novel targets, non-lethal targets, host-directed therapy

## Abstract

The ever-increasing incidence of drug-resistant *Mycobacterium tuberculosis* infections has invigorated the focus on the discovery and development of novel treatment options. The discovery and investigation of essential mycobacterial targets is of utmost importance. In addition to the discovery of novel targets, focusing on non-lethal pathways and the use of host-directed therapies has gained interest. These adjunctive treatment options could not only lead to increased antibiotic susceptibility of *Mycobacterium tuberculosis*, but also have the potential to avoid the emergence of drug resistance. Host-directed therapies, on the other hand, can also reduce the associated lung pathology and improve disease outcome. This review will provide an outline of recent opportunities.

## 1. Introduction

Tuberculosis (TB), an infectious disease caused by the bacillus *Mycobacterium tuberculosis* (*Mtb*), poses a major threat to public health. In its most recent report, the World Health Organization (WHO) recorded 10.0 million incident cases, which is equivalent to 133 cases per population of 100,000. Worldwide, TB also resulted in 1.6 million deaths and was ranked as the leading cause of death due to a single infectious pathogen, even surpassing the HIV/AIDS epidemic [1]. Furthermore, TB, like other bacterial diseases, is confronted by problems of emerging drug resistance, which places an enormous strain on the public healthcare system [1]. This review provides an outline of opportunities for overcoming anti-TB drug resistance by targeting either novel essential targets, non-essential pathways, or host responses.

## 2. Antimycobacterial Drug Resistance

Antimycobacterial drug resistance emerged with the use of the first effective anti-TB agent streptomycin, discovered in 1944. Many individual TB patients receiving streptomycin improved during the first months of treatment, only to relapse again as treatment continued. It was soon understood that this was due to the evolution of streptomycin-resistant *Mtb* strains. As a result, streptomycin monotherapy was quickly abolished, and the first combination therapy was established by adding *para*-aminosalicylic acid to the treatment. During the subsequent years, different anti-TB agents were introduced and added to the growing multidrug anti-TB regimen. Nowadays, the treatment regimen, recommended by the WHO, compromises a 2-month initiation phase with a cocktail of four first-line anti-TB agents, i.e., isoniazid, rifampicin, ethambutol, and pyrazinamide, followed by a 4-month continuation phase with isoniazid and rifampicin. Despite the early establishment of anti-TB combination therapy, antimycobacterial drug resistance continues to emerge. A crucial factor is patient adherence, which is negatively impacted by the lengthy treatment duration, the complexity of the regimen, and the association of many adverse drug effects. Furthermore, differences in the quality of public health systems and the availability of high-quality anti-TB drugs also contributed to the spread of drug-resistant *Mtb* strains [2]. For some low-incidence countries, the recent increase in the number of asylum seekers and other migrants from TB-endemic countries impacted drug-resistant TB epidemiology as well [3,4]. Worldwide, an estimated 3.5% of new cases and 18% of previously treated cases were multidrug-resistant (MDR), defined as TB resistant to the two most powerful anti-TB drugs isoniazid and rifampicin [1]. Over the years, drug-resistant TB alarmingly rose and even progressed to extensively drug-resistant (XDR), and, most recently, to total drug-resistant (TDR) TB. The former encompasses strains that are not only resistant to at least isoniazid and rifampicin, but are also resistant to second-line anti-TB drugs, i.e., at least one of the fluoroquinolone drugs and one of the injectable aminoglycosides [1]. In case of TDR TB, resistance to a number of drugs beyond the XDR TB definition, including resistance to all clinically recommended drugs, occurs [1]. Due to uncertain defining criteria, the WHO has not officially recognized these TDR strains yet. Nonetheless, the available treatment options for MDR and XDR TB are limited, more expensive, toxic and less effective than those for drug susceptible TB [1].

**Figure 1:**
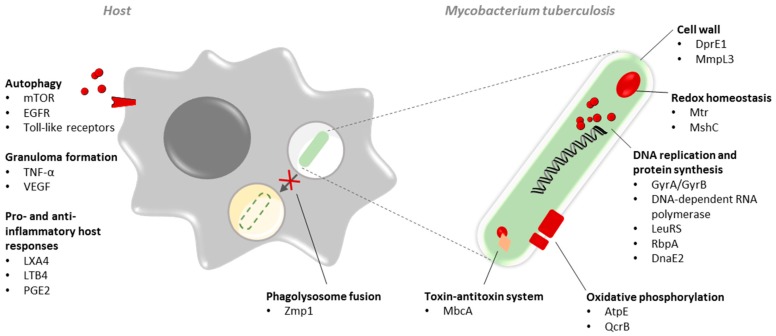
Overview of the emerging mycobacterial targets and host-directed therapies addressed. At the level of *Mycobacterium tuberculosis*, lethal and non-lethal targets can be found in several interesting pathways including, but not limited to, cell wall biosynthesis, redox homeostasis, DNA replication and protein synthesis, oxidative phosphorylation, toxin–antitoxin system and proteins important for phagosome–lysosome fusion. At the level of the host, targeting important processes such as autophagy, granuloma formation, and pro- and anti-inflammatory responses are invigorated to address and overcome drug resistance.

These resistant strains represent a critical threat to global health, prompting the development of alternative therapeutic approaches [1]. Exploring novel mycobacterial drug targets is imperative to effectively address anti-TB drug resistance and improve general TB control in the near future [1]. The development of anti-TB drugs with a novel mechanism of action can be promising as it provides an alternative treatment strategy for resistant TB infections, whereas it could counteract the emergence of drug resistance in combination with existing anti-TB drugs. On the other hand, non-lethal compounds targeting a virulence or stress tolerance factor could render *Mtb* more susceptible to existing or emerging anti-TB chemotherapy due to synergistic effects. As a result, this strategy can overcome the development of drug resistance as well. Alternatively, host-directed therapy (HDT) can also pose an attractive strategy given the delicate balance between *Mtb* and the immunity of its host. Joint efforts of academia, institution bodies, funding bodies, and industry are contributing to the discovery and development of novel anti-TB drugs. The pipeline for TB is steadily filling up, and new anti-TB drug candidates emerge. Along the way, novel mycobacterial drug targets are discovered. At present, many pathways are recognized as promising and pertinent to drug targets. These include those involved in classical cell wall metabolism, cellular respiration, and protein synthesis, although several other points of engagement, such as the bacillary redox homeostasis and toxin–antitoxin systems, could likely provide interesting mycobacterial drug targets. In addition to the discovery of novel targets, focusing on non-lethal pathways and the use of adjunctive HDT is invigorated. This review will address some of the most promising findings (Figure 1).

## 3. Novel Mycobacterial Drug Targets

### 3.1. Cell Wall Biosynthesis

The unique mycobacterial cell wall is comprised of three major covalently linked macromolecules: peptidoglycan, arabinogalactan, and mycolic acids [5]. The pathways responsible for biosynthesis of these major macromolecules particularly provide an important source of mycobacterial targets since the enzymes involved in these pathways do not have homologues in the human host. Cell wall biosynthesis already showed to be a successful pathway for the development of therapeutics as currently used anti-TB drugs include inhibitors of the mycolic acid biosynthesis (isoniazid and ethionamide) [6], arabinan synthesis (ethambutol) [7], and peptidoglycan (cycloserine) [8]. Discoveries of novel mycobacterial targets involved in the assembly mechanisms of the unique *Mtb* cell wall, however, still emerge (Table 1).

These targets include the essential membrane-associated enzyme decaprenylphosphoryl-β-d-ribose-2′-oxidase (DprE1), which is encoded by *dprE1* (*Rv3790*) and designated as the mycobacterial drug target of a novel class of anti-TB agents, i.e., the 1,3-benzothiazin-4-ones (BTZs) [9]. DprE1 belongs to the family of vanillyl alcohol oxidases, a family of FAD-dependent oxidoreductases [10]. Biochemical studies show that DprE1 and decaprenylphosphoryl-2-keto-β-d-*erythro*-pentose reductase (DprE2), encoded by *dprE1′*s neighboring gene *dprE2* (*Rv3791*), catalyze the two-step epimerization of decaprenylphosphoryl-β-d-ribofuranose (DPR) to decaprenylphosphoryl-β-d-arabinofuranose (DPA), a key precursor that serves as the sole arabinosyl donor required for the synthesis of cell wall components arabinogalactan and lipoarabinomannan [9,11]. During development of the BTZs, BTZ043 showed to be the most promising anti-TB drug candidate of this group of nitroaromatic compounds, with a minimal inhibition concentration 20-fold less than that of the first-line agent isoniazid [9,10]. For its mechanism of action, the electron-deficient nitroaromatic BTZ043 is first reduced to an electrophilic nitroso derivative, mediated by the FADH_2_-containing form of DprE1 [12]. Then, the corresponding nitrosoarene covalently reacts with an essential cysteine residue, i.e., Cys387, in the substrate-binding domain of DprE1 to form a stable semimercaptal adduct. This event will irreversibly inactivate the epimerization of DPR to the keto intermediate decaprenylphosphoryl-d-2′-keto-*erythro*-pentofuranose (DPX) and thus disrupt arabinose production [10]. The reduction in arabinogalactan, which acts as a covalent linker between peptidoglycan on the inside and the characteristic mycolic acids at the outer surface, results in reduced cellular integrity [9]. Due to the essentiality of DPA and lack of alternative synthetic pathways [13,14], the novel antimycobacterial drug target DprE1 paved the way for the discovery of other novel anti-TB drug classes able to tackle this intriguing target. In an effort to improve the *in vivo* antimycobacterial activity of the potent BTZ043 suicide inhibitor, a second family of novel anti-TB compounds was discovered. While the mechanism of action was identical, the piperazine-containing BTZs (PBTZs) showed superior properties. The next-generation PBTZ169 proved to be the most suitable candidate for development [15] and emerged alongside BTZ043 as a promising anti-TB candidate effective against both drug susceptible and resistant TB strains, including those resistant to ethambutol. Noteworthy, a missense mutation in the *dprE1* gene, generated by a single point mutation in which Cys387 is replaced by Ser or Gly codons, results in resistance to the covalent DprE1 suicide inhibitors Mutants are rare, though, arising at a frequency of <10^−8^ [9]. Apart from the covalent DprE1 inhibitors BTZ043 and PBTZ169 currently present in the TB drug pipeline, non-covalent DprE1 inhibitors were discovered as well. Two of these alternative DprE1 inhibitors are present in the anti-TB drug pipeline: the 1,4-azaindole TBA7371 and 1,3-dihydrocarbostyril derivate OPC167832, which is being developed by Otsuka Pharmaceutical [16]. Both candidates showed promising anti-TB activity but lack the ability to form a covalent adduct with DprE1. Additionally, this non-covalent inhibition may overcome potential toxicities and resistance associated with the covalent DprE1 inhibitors [17,18,19].

Apart from arabinan synthesis, targets within the assembly mechanisms of mycolic acids are discovered as well. The mycobacterial membrane protein large (MmpL) family of proteins belongs to a group of proton-dependent efflux pumps that mediate the active transport of a diverse array of ionic or neutral compounds as well as various drugs, heavy metals, fatty acids, bile salts, solvents, detergents, and dyes [20,21,22]. The putative MmpL3 transmembrane transporter, encoded by *Rv0206c*, is reported to play a role in heme acquisition by *Mtb* [23], but is more recently designated as the mycobacterial drug target of SQ109 [24]. SQ109, a novel anti-TB agent structurally based on the 1,2-ethylenediamine pharmacophore of ethambutol [25,26] showed advanced *in vitro* and *in vivo* antimycobacterial activity, favorable pharmacokinetic properties, and accumulation in the lung [25,27,28,29]. The activity of SQ109 against drug-resistant strains of *Mtb*, including ethambutol-resistant strains, suggested S109 is a new anti-TB agent rather than an ethambutol analogue [25], and was shown to inhibit the activity of the MmpL3 transmembrane efflux system [24]. Congruently, this observation also resulted in an improved insight in the function of this essential transmembrane transporter. MmpL3 is found to be the unknown transmembrane transporter of trehalose monomycolate (TMM), a non-covalently associated “capsular” lipid which is also involved in the formation of trehalose dimycolate (TDM), or cord factor, and gives rise to the abundant layer of mycolic acid esters present in the outer layer of the closely packed, non-permeable barrier of the waxy cell wall [24,30,31,32,33]. Inhibition of MmpL3 by SQ109 causes TDM depletion along with concomitant upregulation of TMM levels, which ultimately leads to reduced cellular integrity. Treatment with SQ109 results in mycobacterial cell shortening and widening similar to that seen with ethambutol and isoniazid, which respectively affect the assembly of the arabinan an mycolate components of the cell wall [24]. Although MmpL3 was identified as the target of SQ109, the manner of inhibition, i.e., directly or through an indirect mechanism, remained to be determined. Recent studies showed that SQ109 and several other MmpL3 inhibitors, including the indolcarboxamides and diarylpyrroles, act as uncouplers, collapsing the proton motive force (Δp) of the MmpL3 proton antiport mechanism of active efflux rather than interacting directly with the MmpL3 protein [34,35]. It has been reported that the disruptive effect of SQ109 on the proton motive force (Δp) impacts the transmembrane proton concentration gradient (ΔpH), membrane potential (Δψ), or both simultaneously, decreasing ATP synthesis used to power MmpL3 activity [34,36]. Additionally, it is suggested that SQ109 also inhibits enzymes involved in menaquinone synthesis and, hence, are involved in respiration/electron transfer [36]. The development of potent compounds that inhibit multiple targets of general interest are of great importance in the context of developing anti-TB candidates that are resilient to resistance due to multitargeting. Nonetheless, MmpL3 could also provide an excellent target for the development of novel single-target anti-TB drugs, active against both drug susceptible and resistant TB, as it is highly conserved in several mycobacterial species and is demonstrated to be the only member of this MmpL protein family that is essential for *Mtb* replication and viability [37].

Other examples of multitarget anti-TB candidates which inhibit cell wall biosynthesis pathways include the nitroimidazoles pretomanid (PA-824) and delamanid (OPC-67683). Both compounds exert antimycobacterial activity upon metabolization within *Mtb* [38,39]. It is reported that deazaflavin (or cofactor F_420_)-dependent nitroreductase (Dnd), encoded by *Rv3547*, acts as the key catalytic enzyme involved in activating both prodrugs. Although its exact role in *Mtb* is not yet clear, Dnd is proposed to be an accessory protein of the coenzyme F_420_-dependent glucose-6-phosphate dehydrogenase (FGD1), which is in turn responsible for the reduction of deazaflavin cofactor F_420_. Recruiting the necessary electrons for bioreductive activation, provided by FGD1 or its coenzyme F_420_, Dnd is able to directly interact with pretomanid and delamanid, hereby metabolizing their nitro residue and causing the formation of radical intermediates [39,40,41]. Activated prodrugs are assumed to target replicating *Mtb* by the inhibition of mycolic acid and mycobacterial protein synthesis. It is shown that pretomanid inhibits a metabolic step responsible for the oxidation of hydroxymycolate, a precursor of the essential ketomycolate [38,42]. In contrast to the current anti-TB agents, the nitroimidazoles also exhibit bactericidal activity against non-replicating persistent *Mtb* [38,43]. In non-replicating bacilli, the bactericidal activity likely stems from the pleiotropic action of nitroimidazole-derived radicals, which could poison the bacterial respiratory chain and result in ATP depletion [38,40,42]. Both pretomanid and delamanid have the potential to contribute to MDR and XDR TB treatment due to the apparent lack of cross-resistance with a variety of other anti-TB drugs which also confirms a novel mechanism of action for both compounds [38,42,43], although resistance to both anti-TB candidates has been demonstrated. The rate of spontaneous resistance against pretomanid in *Mtb* ranges from 10^−7^ to 10^−8^, approaching that of isoniazid, another prodrug that requires a non-essential mycobacterial activating system [38,40]. Consistently, resistant mutants show genetic modifications within the genes involved in the prodrug bioactivating pathway, i.e., the gene encoding for FGD1, coenzyme F_420_ biosynthesis pathway-related proteins (FbiA, FbiB, and FbiC) and the accessory protein Dnd, the key enzyme involved in nitroimidazole activation [39,40,41,42,44]. The same mutations have found to be the cause of resistance against delamanid [39,45].

### 3.2. Oxidative Phosphorylation

Oxidative phosphorylation is the central mechanism of energy production for mycobacteria. Here, electrons derived from the oxidation of both organic and inorganic substrates are transferred through a series of complexes, known as the electron transport chain (ETC), to reach electron acceptors. This electron transport chain includes type II NADH dehydrogenase, responsible for feeding electrons derived from NADH into the transport chain, leading to the reduction of the menaquinone pool. Alternatively, the menaquinone pool can also be reduced by alternative electron donors, e.g., via the succinate dehydrogenase. From the menaquinone pool, electrons are then transferred to the cytochrome *bc_1–_aa_3_* supercomplex which, in turn, transfers the electrons onto oxygen. Oxygen can also be reduced by a cytochrome *bd*-type terminal oxidase, accepting electrons directly from the menaquinone pool. The movement of electrons through these complexes in turn enables the transfer of protons across the membrane (ΔpH and Δψ), leading to a proton motive force (Δp). The energy of the electrochemical proton gradient then drives the final step, i.e., synthesis of ATP by ATP synthase. As *Mtb* is an obligate aerobic pathogen, the process of oxidative phosphorylation is essential for mycobacterial survival and growth [46,47]. Due to its essential character, the oxidative phosphorylation makes up an interesting source of novel targets for anti-TB drug discovery (Table 1).

One of the most striking examples includes the novel antimycobacterial target represented by ATP synthase, encoded by the *atpBEFHAGDC* operon (*Rv1303-1312*) [48,49,50]. The ubiquitous enzyme, important for the energy metabolism of mycobacteria, is composed of two structural domains, i.e., a membrane-embedded F_0_ part and a hydrophilic F_1_ part [48,49]. The passage of ions through the F_0_ transmembrane channel triggers the rotary catalysis of the F_1_ extramembranous catalytic core, resulting in the synthesis of ATP [49,51]. Due to its essentiality in both replicating and non-replicating conditions [13,49], ATP synthase represents a highly attractive target for the development of novel anti-TB candidates. Bedaquiline (BDQ), formerly known as R207910 or TMC207, belongs to the chemical class of the diarylquinolines (DARQ) and acts as an inhibitor of the ATP synthase c subunit, encoded by *atpE* (*Rv1305*) [50,52,53,54]. It is proposed that binding of BDQ to the c-ring perturbs the a–c subunit interface, resulting in an uncontrolled proton leak that is uncoupled from the ATP synthesis. Eventually, this action will lead to an ineffective proton cycle, which is lethal to mycobacteria [55]. Other studies suggest that BDQ can also bind to the subunit ε or can interfere with the efflux of small molecules [52,56,57]. BDQ lacks the display of cross-resistance with currently used anti-TB agents, highlighting its substantial influence on MDR and XDR TB treatment [53]. Therefore, BDQ received a fast-track approval by the US Food and Drug Administration and the European Medicines Agency for treatment of multidrug-resistant tuberculosis, and is hereby the first anti-TB compound to be authorized for use in humans in over 40 years [52,55]. However, reports of BDQ resistance emerged soon after its introduction, with a mutation frequency ranging from 10^−7^ to 10^−8^. Currently known mechanisms of BDQ resistance do not only include the introduction of point mutations within *atpE*, but also involve non-target-based mutations in *Rv0678*, a transcriptional repressor of the genes encoding the mycobacterial membrane protein small (MmpS)5-MmpL5 efflux pump, and *pepQ*, a gene with unclear function [46,53,58,59]. In the attempt to improve the drug-likeliness of BDQ without altering its anti-tubercular activity, other DARQ analogues were synthesized and evaluated. Within this framework, TBAJ-587 and TBAJ-876 were selected for preclinical development due to their increased *in vitro* effect against *Mtb* clinical isolates, *in vivo* efficacy against murine TB at lower exposures, lower potency against the *human ether-a-go-go-related* gene (*hERG*)-encoded cardiac potassium channel, predicted higher human clearance, and acceptable safety margin. The structure of the TBAJ analogues differs from that of BDQ as it contains a 3,5-dialkoxy-5-pyridyl group instead of the C-unit naphthalene ring, whereas the mechanism of action remains the same. Acquired resistance is also associated with mutations in the *atpE* and *Rv0678* genes [46,60]. By contrast, a recent high-throughput screening enabled the identification of a new class of specific and selective ATP synthase inhibitors, i.e., the squaramides. Although their mode of action is similar to that of BDQ, they are thought to have another binding site on the ATP synthase enzyme as cross-resistance against BDQ-resistant mutants was absent. Therefore, the squaramides are also found to be promising candidates for the development of novel anti-TB therapeutics [61].

Another point of engagement was recently discovered via a phenotypic high-content screening against *Mtb* inside macrophages. The study has led to the discovery of Q203, an imidazopyridine amide potently targeting the respiratory cytochrome *bc*_1_ complex [62,63]. This complex, also referred to as ubiquinol–cytochrome *c* oxidoreductase, plays an important role in the mycobacterial respiratory chain. It functions through transferring reducing equivalents from a low-potential quinol to a high-potential acceptor protein, i.e., cytochrome *c*, generating proton translocation across the membrane [64,65]. Q203 is found to bind the *b* subunit QcrB of this essential complex, encoded by *qcrB* (*Rv2196*), disrupting the mycobacterial respiratory chain and rapidly leading to ATP depletion in *Mtb* grown under aerobic and anaerobic conditions [62,63]. Due to its novel mechanism of action, Q203 is also found to be effective against MDR and XDR *Mtb* strains [62]. In contrast, resistance against Q203 can be obtained by the generation of a single point mutation in *qcrB*, whereby Thr313 is replaced by Ala or Ile codons. Though, it is concluded that the generation of spontaneous mutants is rare, arising at a frequency of approximately 2.4 × 10^−8^ [62]. These interesting observations paved the way for the discovery of other novel anti-TB drug classes able to tackle this intriguing mycobacterial target, such as the pyrrolo [3,4-c]pyridine-1,3(2*H*)-diones, 2-(quinolin-4-yloxy)acetamides and arylvinylpiperazine amides. Interestingly, AX-35, which belongs to the latter compound class, seems to inhibit QcrB via a different mode of action than the other QcrB inhibitors. Further research will focus on the optimization of these compounds into proper anti-TB candidates [64,66].

### 3.3. Protein Synthesis

Protein biosynthesis is a fundamental requirement for survival and replication of all cells, including *Mtb*, though the biological processes responsible can differ between organisms, indicating that they provide an important source of (novel) mycobacterial targets [67]. Mycobacterial gene expression and eventual protein biosynthesis have already been shown to be vulnerable to chemotherapeutic intervention. Currently used anti-TB drugs include inhibitors of the DNA unwinding process necessary for replication and protein synthesis (fluoroquinolones) [68], gene transcription by DNA-dependent RNA polymerase (rifampicin) [69], and ribosomal translation (streptomycin, amikacin, kanamycin and capreomycin) [70]. Besides the discovery of novel points of engagement, the discovery of distinct active sites on well-validated antimycobacterial targets within gene expression and protein biosynthesis processes also provides an interesting strategy for the development of novel anti-TB candidates (Table 1).

A primary example of this interesting strategy is featured within the process of DNA unwinding. During this key step, type II DNA topoisomerase modulates the mycobacterial chromosome topology by performing a transient double-stand DNA cleavage, either relaxing supercoils, decatenating, or introducing negative DNA supercoils. The fluoroquinolones, a highly successful group of second-line anti-TB agents, target the active site for DNA-cleavage of the DNA gyrase A domain (GyrA). As a result, DNA relegation is disrupted and the enzyme–DNA complex is converted into a DNA-cleavage complex, releasing highly lethal double-strand DNA breaks. Despite the attractive form of dual targeting of these highly related mycobacterial type II topoisomerases, the rise of clinical fluoroquinolone-resistance has compromised the use of this important class of anti-TB agent in MDR TB treatment. Recently, a novel series of DNA gyrase inhibitors, i.e., the thiophenes, were identified. These inhibitors are able to bind to a site on the DNA gyrase enzyme distinct from the known fluoroquinolones, resulting in a different mode of action and the absence of cross-resistance. Whereas the fluoroquinolones work by blocking DNA gyrase at the point to which it interacts with DNA, the thiophenes rather bind remotely from the DNA, indicating an allosteric mechanism for DNA-cleavage complex stabilization. Furthermore, the frequency of thiophene resistance was found to be low, suggesting either that the residues involved in binding of the thiophenes are important for enzyme function and/or that the novel binding pocket is small enough that the number of mutable residues is limited. Although the two most successful compounds of this novel series of DNA gyrase inhibitors could not be optimized into developable anti-TB candidates, the discovery of novel molecules against well-validated targets is highlighted as an interesting strategy for delivering new candidates for clinical trials. Moreover, the unexploited thiophene-binding pocket potentially provides an interesting mycobacterial target for the development of novel anti-TB candidates [71]. Alternatively, the validated DNA gyrase could also be inhibited by targeting the energy supply DNA gyrase B domain (GyrB) of the enzyme. Recently, SPR720 (formerly known as VXc-486), an aminobenzimidazole analogue, was reported to potently inhibit the ATP synthase activity conferred by the DNA GyrB subunit, abolishing the energy-dependent reactions catalyzed by DNA gyrase. As fluoroquinolone-resistance is acquired via the generation of point mutations in the DNA GyrA domain, the discovery and development of GyrB inhibitors could offer a tremendous opportunity for the treatment of MDR and XDR TB [72,73].

Further, DNA transcription, ultimately allowing translation and mycobacterial protein biosynthesis, not only includes a well-validated antimycobacterial target that could possibly be inhibited in an alternative fashion, but could also serve as a potential source of new anti-TB targets. This key process is initiated by DNA-dependent RNA polymerase. It operates as a complex molecular machinery sharing extensive interactions with the DNA template and the product RNA, regulated through interaction with a series of transcription factors [69,74]. The β subunit, encoded by *rpoB*, forms the primary binding site of the antimycobacterial rifamycins, including the first-line anti-TB agent rifampicin. Binding of rifampicin to the RNA polymerase–DNA complex physically blocks the early phase of extension of the growing oligonucleotide chain [69]. Due to the fact that mycobacterial DNA-dependent RNA polymerase is not similar to its eukaryotic homolog, the enzyme forms an excellent target [74]. Although the β subunit of mycobacterial RNA polymerase embodies the primary target of a first-line anti-TB agent, it is plausible many distinct sites exist representing excellent targets for the discovery of novel anti-TB candidates which could disrupt critical features of the functional mechanism [69,74,75]. For example, the non-rifampicin related Nα-aroyl-*N*-aryl-phenylalaninamides (AAPs) bind to a different site on *Mtb* DNA-dependent RNA polymerase than rifampicin, inhibiting *Mtb* growth. The AAPs exhibit no cross-resistance with rifampicin, function additively when co-administered with rifampicin, and suppress the emergence of rifampicin drug resistance. Together with an extended knowledge of RNA polymerase structure and function, these preclinical lead compounds pave the way for the discovery and development of novel anti-TB candidates targeting this well-validated target [75]. Secondly, the indispensable RNA polymerase-associated transcription factors, which are not conserved in eukaryotic host cells and essential for mycobacterial viability, remain to be an underutilized target [69,74].

Another attractive mycobacterial target required for protein synthesis includes the essential aminoacyl-tRNA synthases (AARSs) family of enzymes. Leucyl-tRNA synthase (LeuRS) is a class I AARS with two separated active sites: a synthetic site that aminoacylates tRNA^Leu^, and an editing site that ensures the fidelity of translation by a proofreading mechanism. Recently, a series of oxaboroles were identified as potential LeuRS inhibitors. These boron-containing compounds have been shown to act on the editing site of LeuRS and disrupt its proofreading role in leucyl-tRNA synthesis. GSK656, the most promising candidate, traps tRNA^Leu^ in the editing site in a nonproductive complex via the formation of a covalent adduct between the 3′ end ribosyl diols and the boron atom, ultimately leading to the inhibition of leucylation and, thus, protein synthesis [76,77]. Other AARSs, including aspartyl-tRNA synthase and tyrosyl-tRNA synthase, have also been studied [78,79].

### 3.4. Bacillary Redox Homeostasis

Apart from the cell wall metabolism, cellular respiration and mycobacterial protein synthesis, the redox homeostasis also makes up an interesting target for the development of novel anti-TB candidates. *Mtb* is not only subjected to endogenous reactive oxygen species (ROS) as part of normal aerobic respiration, it is also continuously exposed to harmful exogenous ROS and reactive nitrogen species (RNS) generated by the host’s immune system in order to clear invading pathogens. Furthermore, *Mtb* encounters a range of environmental oxidants and reductants, found within the different microenvironments during its course of infection, as well. These could all disrupt the intracellular oxidation–reduction balance that is important for *Mtb* survival, persistence, and reactivation. In order to monitor redox alterations and maintain its desired redox balance, *Mtb* evolved various pathways [80].

One of these pathways includes the mycothiol (MSH) biosynthesis and recycling pathway. This pathway is reported to be a promising source of novel mycobacterial targets as it is unique for the phylum of *Actinobacteria*, a group of Gram-positive bacteria to which *Mtb* belongs. MSH or acetylcysteine-glucosamine-inositol (AcCys-GlcN-Ins), a low-molecular-weight thiol, plays a crucial role in offering a first line of protection against endogenous and exogenous ROS and RNS [81,82]. Furthermore, MSH is also reported to be indispensable for the detoxification of oxygen stress through the action of several anti-TB agents, such as isoniazid, and to serve as a co-factor in many regulatory processes [81,83]. The biosynthesis of MSH proceeds via a five-step process involving 5 different enzymes, including cysteine ligase MshC and mycothione reductase (Mtr). The penultimate step of the MSH biosynthesis is performed by MshC, in which it catalyzes the ligation of glucosamine-inositol (GlcN-Ins) to cysteine in order to form Cys-GlcN-Ins. Acting as a scavenger, MSH captures ROS and becomes oxidized to form mycothione (or mycothiol disulfide; MSSM). To maintain a reductive environment within the bacillus, a constant MSH/MSSM ratio is maintained by the recovery of MSH form MSSM by Mtr [84,85,86]. MshC and Mtr are both found to be promising mycobacterial targets as they are highly druggable and unique for the *Actinomycetes*, thereby limiting the chance of hitting off-targets within the host or the host’s microbiome and reducing the possibility of side effects (Table 1) [83,87]. Additionally, it is suggested that both enzymes are essential for *Mtb* replication and viability [13,88,89]. However, this statement has not been confirmed by further research, which is assumed to be due to the lack of a phenotypic benchmark of the enzymes. Furthermore, Mtr and MshC are especially attractive targets because there likely is a synergistic activity between possible Mtr and MshC inhibitors and other anti-TB candidates still under preclinical and clinical development. Many of these anti-TB candidates, including BDQ and Q203, will cause respiratory poison by ROS due to the inhibition of the ETC. Hereby, inhibition of Mtr and MshC will prevent *Mtb* to react to the respiratory poison, resulting in faster kill kinetics. For this reason, potential Mtr and MshC inhibitors could present the ideal companion drugs for the current leads in (pre-)clinical development and could lead to a significant reduction in the required treatment length [46]. So far, no specific inhibitors have been identified for Mtr or MshC. Although dequalinium was reported to be an enzymatic inhibitor of MshC, as an antiseptic and disinfectant, its use is limited to a topical application [90]. Furthermore, some naphthoquinone derivatives like 7-methyljuglone have been investigated as potent subversive substrates for Mtr in the past, but they have shown to be equally reactive with glutathione reductase leading to non-specific compounds with severe cellular toxicity [91]. Similarly, the closely related benzo[*g*]isoquinoline-5,10-diones and benz [*g*]phenanthridine-7,12-diones have been investigated as well [92,93]. In order to reduce the cytotoxic effects of the latter group, which possibly arose from the intercalating effects of these rather planar structures, various substituted “out of plane” tetrahydrobenzo[*j*]phenanthridine-7,12-diones and octahydrobenzo[*j*]phenanthridine-7,12-diones are under investigation [94].

### 3.5. Toxin–Antitoxin System

More recently, the *Mtb* toxin–antitoxin (TA) system has been brought to attention in the context of discovering novel anti-TB treatment options. These TA systems are widely distrusted in prokaryotic genomes and regulate fundamental cellular processes in the response and adaptation of bacteria to various stress conditions, such as starvation, phage attack, environmental stress, or antibiotic treatment. In the *Mtb* genome, an expanded repertoire of TA system-encoding loci have been identified. Under favorable growth conditions, the toxic protein, which reduces mycobacterial growth rate, is neutralized by the presence of the interacting antitoxin protein. When exposed to unfavorable conditions, the antitoxin is rapidly degraded, allowing the toxin to cause growth inhibition [95].

Among the TA system encoding two-gene operons identified in the *Mtb* genome, three antitoxin-encoding genes are essential for *Mtb* viability. This suggests that their toxin counterparts could induce a lethal effect rather than a growth-inhibiting effect. Therefore, these TA systems could illuminate a novel approach to treat both drug susceptible as well as resistant TB infections. Of late, the new mycobactericidal toxin (MbcT) and antitoxic (MbcA) system (*Rv1989c*–*Rv1990c*), which is upregulated in several conditions—including hypoxic stress and starvation in *Mtb* persister cells and within host macrophages—is proposed as interesting point of engagement. MbcT is elucidated as a highly efficient NAD^+^ phosphorylase, responsible for the catalyze of NAD^+^ degradation *in vitro* and *in vivo*. If not opposed by its MbcA antitoxin, the “suicide” MbcT toxin will rapidly kill *Mtb* bacilli as NAD^+^ depletion is lethal in mycobacteria. Small molecule inhibitors that disrupt this MbcTA complex or inactivate its essential MbcA antitoxin represent potential therapeutics of interest [96].

## 4. Non-Essential Targets

As one of the most successful human pathogens, *Mtb* employs several mechanisms to facilitate survival upon antibiotic stress. Besides its intrinsic robustness which allows *Mtb* to survive in the presence of anti-TB drugs for long enough to develop resistance, *Mtb* is able to generate persisters that can survive high concentrations of anti-TB drugs without the induction of genetic modifications. The development of these drug-tolerant *Mtb* bacilli is not only stimulated by antibiotic stress, but also by environmental stresses such as starvation, hypoxia, or low pH. Addressing pathways underlying the formation of drug-tolerant persisters, which are non-lethal per se, could influence stress responses and render *Mtb* to be more susceptible to both existing and novel anti-TB agents designed to hit lethal targets (Table 2).

In the search to find appropriate adjuvant-therapy targets, the non-essential DNA-dependent RNA polymerase binding protein RbpA was discovered. As RNA polymerase-associated transcription factors are, as mentioned earlier, indispensable for the initiation of gene transcription, RbpA could serve as an interesting mycobacterial target. It is suggested that the RbpA RNA polymerase binding protein regulates the activities of the principal housekeeping sigma factor σ^A^ and the stress response σ^B^ in mycobacteria, which is showed to be involved in tolerance to both oxidative and antibiotic stress. A recent study indicated that the association of RbpA with σ^B^ activated the transcription of *ppk1*, which encodes polyphosphate kinase. As a result, intracellular levels of inorganic polyphosphate increase, promoting isoniazid-tolerant mycobacteria [97]. These data suggest that the development of novel chemical entities interrupting this isoniazid-tolerance promoting pathway could, when co-administered, improve isoniazid function and potentially suppress the emergence of isoniazid drug resistance.

Another interesting example of non-lethal targets includes the replicative DNA polymerase DnaE2 enzyme. The mycobacterial DnaE2, encoded by *Rv3370c*, belongs to the C family of error-prone DNA polymerases. Although DnaE2 seems to be non-essential for chromosomal replication, it is thought to play an important role in the *in vivo* survival and drug resistance of *Mtb*. When *Mtb* is being subjected to ROS and RNS produced by the host’s immune response, DnaE2 will directly contribute to the generation of mutations within the mycobacterial genome. This phenomenon may be responsible for the generation of mycobacterial mutants that are better adapted for *in vivo* survival and/or antibiotic stress. Otherwise, when DnaE2 activity is lost, a decreased frequency of drug-resistant *Mtb* mutants is observed as *Mtb* becomes hypersensitive to DNA damage, whereas induced mutagenesis is eliminated [98,99,100]. Due to the fact that DnaE2 has no significant human analogues, the error-prone DNA polymerase would form an excellent mycobacterial target as off-target inhibition would be limited [101]. Recent in silico screening has identified two 6-anilino-1*H*-pyrimidine-2, 4-dione analogs which were found to bind DnaE2 with high affinity, and are thought to inhibit the replicative activity of this mycobacterial enzyme. As DnaE2 inhibition will render *Mtb* more sensitive to the host’s immune system and to anti-TB chemotherapy, these inhibitors may function as interesting companion drugs [101].

Next, the putative Zn^2+^ metalloprotease (Zmp1), encoded by *Rv0198c*, also forms an attractive target [102,103]. Although its exact mechanism of action has yet to be unraveled, its role in *Mtb* pathogenicity has been confirmed. Zmp1 is thought to interfere with the host’s immune system through the prevention of inflammasome activation by inhibiting caspase-1 activation and processing of pro-interleukine (IL)-1β into Il-1β. This process leads to the suppression of phagosome maturation, which demonstrates the important role of Zmp1 for *Mtb* survival inside macrophages [102]. Another study suggested that Zmp1 is also responsible for mycobacterial hypervirulence in mice [104]. Alternatively, Zmp1 deletion is shown to increases the immunogenicity of *Mycobacterium bovis* BCG, the vaccine strain, by enhancing the presentation of mycobacterial antigens [105]. As it is thought that Zmp1 plays an important role in mycobacterial survival, it is considered an attractive target for the development of potential anti-TB adjuvants. Potent inhibitors of Zmp1 are described and include ZTB23(R) [106], arylidene rhodanine derivatives [107] and *N*-(benzyloxy)-8-hydroxyquinoline-2-carboxamide [108].

## 5. Host-Directed Therapy

The current TB treatment is far from ideal and demonstrates several shortcomings, including treatment duration, frequent association of adverse drug effects, and low success rate. In order to overcome these shortcomings and hereby improving anti-TB chemotherapy, the exploitation of novel treatment options is necessary. Over the past years, many studies have been conducted to unravel new potential treatment strategies to fight this deadly disease and overcome the ever-increasing anti-TB drug resistance. Within this framework, HDT has gained considerable interest as it would allow the optimization of TB treatment by shortening treatment length, reducing the number of anti-TB agents required in combination therapy, avoiding the emergence and improving the treatment of drug-resistant TB, and preserving lung function by avoiding extensive tissue damage [109,110].

The course of TB infection is primarily determined by both the *Mtb* pathogen and host immune system, including innate and adaptive defense reactions. *Mtb*, however, has developed mechanisms to evade these protective host immunity reactions at different phases of TB infection [111]. HDTs, however, strive to modulate these host immune responses in order to target specific pathways and/or reduce related symptoms during the course of infection. Hereby, these therapies could stimulate a more efficient response in the fight against *Mtb.* With the aid of small molecules or biologicals, HDT aims to (i) interfere with host mechanisms or pathways that are beneficial for the course of the disease, or (ii) boost the immune system against the pathogen to improve disease clearance. Furthermore, HDT can also play a role in alleviating TB symptoms by targeting pathways or factors associated with TB disease, immunopathology or pathogenic responses [110]. Moreover, HDT can be a useful tool to improve anti-TB treatment efficiency and are less prone to therapy resistance in comparison with anti-TB drugs targeting the pathogen directly. Conferring resistance to a HDT is a difficult task as *Mtb* would need to avoid activated host defense mechanisms, become less dependent of the targeted host factors for replication and survival, or make extensive mutational changes [112,113]. As these molecules primarily modulate host cell functions and can be administered together with other anti-TB agents directly effective against *Mtb*, HDT appears a promising strategy to both avoid anti-TB drug resistance emergence, and in case of pre-existing MDR and XDR TB, effectively tackle these hurdles [110,114]. In the last few years, several molecules and repurposed drugs are proposed to serve these purposes and are further investigated for their exploitation in anti-TB chemotherapy (Table 3).

As the most prominent hallmark of TB disease, i.e., granuloma formation, is driven by both mycobacterial and host factors in the lung, this process could thereby offer promising targets for HDT. For example, the chemokine tumor necrosis factor (TNF)-α plays a key role in granuloma formation and maintenance of its integrity [115]. Neutralization of TNF-α using a TNF-α inhibitor, such as etanercept, is of increased interest as it leads to disruption of granuloma integrity, which in turn augments anti-TB drug response and reduces lung pathology [116,117,118]. Another important characteristic of these fibrotic structures includes the increased expression of vascular endothelial growth factor (VEGF) and angiopoietins that promote abnormal angiogenesis and hypoxic microenvironments [119]. Similar to anti-tumor treatment, the administration of the anti-VEGF antibody bevacizumab promotes vascular normalization and reduces hypoxic fractions. These morphological changes, in turn, facilitate improved penetration and efficacy of current anti-TB drugs [119,120].

Another interesting point of engagement includes the autophagy pathway. In eukaryotic cells, autophagy is the major process of degradation or recycling of cytoplasmic portions or targets. In the case of intracellular pathogens, autophagy is initiated as an innate defense mechanism to restrict replication and survival of the concerned pathogen. This highly regulated and dynamic self-digestion process relies on the maturation of phagosomes into phagolysosomes, via fusion with lysosomes, and the subsequent bulk degradation of the engulfed components [121,122]. Moreover, many autophagy-inducing agents, including interferon (IFN)-γ, were shown to increase both *Mtb* delivery to the autophagosomes and activation of the latter via lysosome fusion and, thus, improve mycobacterial clearance [122,123]. However, *Mtb*, which resides and replicates inside the macrophage’s phagosomes, escapes autophagy by inhibiting phagolysosome fusion and, hereby, increase its survival. Therefore, inducing autophagy would be an appealing HDT strategy to help fight this intriguing pathogen. The best-studied autophagy-inducing compound is rapamycin, an immunosuppressive drug used in organ transplants. Rapamycin inhibits the mammalian target of rapamycin (mTOR), which is a strong negative regulator of autophagy [124]. However, its application as HDT for TB is limited since rapamycin absorption is highly variable and concerning adverse reactions can occur. Moreover, rapamycin is metabolized by the hepatic enzyme CYP3A4, which is strongly induced by the key first-line anti-TB drug rifampicin [109,110,114]. Anticonvulsant drugs, including carbamazepine and valproic acid, were also shown to act as autophagy stimulators, and this through an mTOR-independent pathway [125]. Another manner for autophagy induction includes the inhibition of epidermal growth factor receptor (EGFR)-mediated kinase signaling cascade. Gefitinib, an EGFR inhibitor and, thus, autophagy activator, was shown to be a promising HDT candidate for TB as it is able to decrease mycobacterial growth inside macrophages and murine lungs [126]. The process of autophagy can also be promoted via the activation of human macrophage Toll-like receptors, leading to subsequent induction of cathelicidin and other antimicrobial peptides. As vitamin D is required for their production process, it is considered essential for antimycobacterial host defense and, hereby, an interesting HDT candidate [127,128,129]. Besides upregulating innate immune functions via pleiotropic effects, including autophagy induction, vitamin D also regulates inflammatory host responses by downregulating the production of pro-inflammatory cytokines and chemokines, augmenting the production of anti-inflammatory cytokines and interfering with T cell responses [130,131,132,133].

Due to the fact that the balance between pro- and anti-inflammatory host responses is critical to both mycobacterial control and lung pathology, targeting the inflammatory response via HDT forms another interesting strategy. Host inflammatory balance is, for instance, influenced by the level of lipoxin A4 (LXA4) and leukotriene B4 (LTB4): LXA4 helps to maintain inflammatory balance and is key in the control of TB progression while LTB4 causes hyperinflammation and increases disease severity [134,135]. Therefore, attenuating excessive host inflammatory responses might be beneficial during treatment and may improve disease outcome. Based on its anti-inflammatory character, acetylsalicylic acid was suggested to be a potential HDT candidate. It is shown that acetylsalicylic acid enhances LXA4 production, which in turn suppresses neutrophil migration and TNF-α production. Thereby, it is able to regulate inflammatory lung pathology during *Mtb* infection and improve treatment outcome [134,136,137]. Another influence strategy includes the suppressing of prostaglandin E2 (PGE2), which regulates inflammation and is responsible for inhibiting mycobacterial phagocytosis and killing [135]. Non-steroidal anti-inflammatory drugs (NSAIDs), including diclofenac and ibuprofen, are able to reduce PGE2 induction via inhibition of cyclooxygenase (COX)-1 and COX-2. Preclinical studies suggested they could alleviate inflammatory responses and, thus, lung pathology during TB disease [137,138,139].

## 6. Conclusions

Drug-resistant TB possess a significant challenge for TB control and elimination in many parts of the world. In order to effectively control the TB epidemic, development of novel treatment options for drug-susceptible and -resistant TB is of utmost importance. After almost four decades of neglect, significant advances in the field of drug discovery and development are being made. Due to funding and market incentive mechanisms promoting and supporting anti-TB drug development, several new promising anti-TB candidates for the treatment of drug-resistant TB are in clinical development. Apart from the clinical evaluation of novel analogues of validated anti-TB drug classes, including the riminophenazine analogue TBI-166 [140] as a less toxic alternative of the second-line anti-TB agent clofazimine and the oxazolidinone analogues TBI-223 [141], contezolid [142,143,144], sutezolid [145,146] and delpazolid [147,148] as more effective and safer linezolid alternatives, novel chemical entities are assessed as well. As these candidates interact with novel mycobacterial targets, they may provide an alternative treatment option for drug-resistant TB infections and could counteract the emergence of drug resistance when given in combination with existing anti-TB drugs. New mycobacterial targets are found not only in classical pathways of cell wall metabolism, cellular respiration, and protein synthesis, and lethal targets have also emerged in novel mycobacterial pathways, such as the bacillary redox homeostasis and toxin–antitoxin systems. Another promising strategy to tackle drug-resistant TB includes the discovery and development of non-lethal compounds targeting a virulence or stress tolerance factor. These could render *Mtb* more susceptible to existing or emerging anti-TB chemotherapy when administered in combination and possibly circumvent the development of drug resistance. The identification of several promising adjuvant targets, including RbpA, DnaE2, and Zmp1, have opened the prospects for identifying other non-lethal anti-TB candidates. Finally, HDT also poses an attractive strategy for the treatment of drug-resistant TB as it could promote a faster and more efficient clearance of *Mtb* when combined with less effective second-line anti-TB drugs and improve the general clinical outcome. Meanwhile, in the last few years, several preclinical studies have been conducted, and ongoing and starting clinical trials will help to assess safety and efficacy of HDT candidates. Although significant advances have been made, the challenge in tackling MDR and XDR TB still needs to be efficiently addressed.

## Figures and Tables

**Table 1:** Novel mycobacterial target examples and their relevant compounds or compound classes.

Targeted Pathway	Target	Relevant Compound (Class)
Cell wall biosynthesis	DprE1	BTZ043 (BTZ)
	DprE1	PBTZ169 (PBTZ)
	DprE1	TBA7371 (1,4-azaindole)
	DprE1	OPC167832 (1,3-dihydrocarbostyril)
	MmpL3	SQ109 (1,2-ethylenediamine)
Cell wall biosynthesis and Protein synthesis	-	Pretomanid/PA-824 (nitroimidazole)
	-	Delamanid (nitroimidazole)
Oxidative phosphorylation	AtpE	BDQ (DARQ)
	ATP synthase	Squaramides
	QcrB	Q203 (imidazopyridine)
	QcrB	Pyrrolo[3,4-c]pyridine-1,3(2*H*)-diones
	QcrB	2-(Quinolin-4-yloxy)acetamides
	QcrB	AX-35 (arylvinylpiperazine amide)
Protein synthesis	GyrA	Thiophenes
	GyrB	SPR720 (aminobenzimidazole)
	DNA-dependent RNA polymerase	AAPs
	LeuRS	GSK656 (oxaborole)
Bacillary redox homeostasis	MshC	Dequalinum
	Mtr	Benzo[*g*]isoquinoline-5,10-diones
	Mtr	Benzo[*j*]phenanthridine-7,12-diones

**Table 2:** Non-essential mycobacterial target examples and their relevant compounds or compound classes.

Targeted Pathway	Target	Compound (Class)
Replication	DNAE2	6-anilino-1*H*-pyrimidine-2,4-diones
Phagosome maturation	Zmp1	ZTB23(R)
	Zmp1	Arylidene-rhodanines
	Zmp1	*N*-(benzyloxy)-8-hydroxyquinoline-2-carboxamide

**Table 3:** HDT-related target examples and their relevant compounds or compound classes.

Targeted Pathway	Target	Compound (Class)
Granuloma formation	TNF-α	Etanercept
	VEGF	Bevacizumab
Autophagy	mTOR	Rapamycin
	-	Carbamazepine
	-	Valproic acid
	EGFR	Gefitinib
	Cathelicidin (and others) biosynthesis	Vitamin D ^a^
Pro- and anti-inflammatory host responses	LXA4 production	Acetylsalicylic acid
	COX-1; COX-2	NSAIDs

^a^ Besides upregulating innate immune functions via pleiotropic effects, including autophagy induction, vitamin D also regulates inflammatory host responses by downregulating the production of pro-inflammatory cytokines and chemokines, augmenting the production of anti-inflammatory cytokines and interfering with T cell responses.

## Abbreviations

AAP	Nα-aroyl-*N*-aryl-phenylalaninamide
AARS	aminoacyl-tRNA synthase
AcCys-GlcN-Ins	acetylcysteine-glucosamine-inositol
BDQ	bedaquiline
BTZ	1,3-benzothiazin-4-one
COX	cyclooxygenase
DARQ	diarylquinoline
Dnd	deazaflavin (or cofactor F_420_)-dependent nitroreductase
DPA	decaprenylphosphoryl-β-d-arabinofuranose
DPR	decaprenylphosphoryl-β-d-ribofuranose
DprE1	decaprenylphosphoryl-β-d-ribose-2′-oxidase
DprE2	decaprenylphosphoryl-2-keto-β-d-*erythro*-pentose reductase
DPX	decaprenylphosphoryl-d-2′-keto-*erythro*-pentofuranose
EGFR	epidermal growth factor receptor
ETC	electron transport chain
FGD1	coenzyme F_420_-dependent glucose-6-phosphate dehydrogenase
GlcN-Ins	glucosamine-inositol
GyrA	DNA gyrase domain A
GyrB	DNA gyrase domain B
hERG	human ether-a-go-go related gene
HDT	host-directed therapy
IFN-γ	interferon-γ
IL	interleukine
LeuRS	leucyl-tRNA synthase
LTB4	leukotriene B4
LXA4	lipoxin A4
MbcA	mycobactericidal antitoxin
MbcT	mycobactericidal toxin
MDR	multidrug resistant
MmpL	mycobacterial membrane protein large
MmpS	mycobacterial membrane protein small
MSH	mycothiol
MSSM	mycothione or mycothiol disulfide
mTOR	mammalian target of rapamycin
Mtr	mycothione reductase
*Mtb*	*Mycobacterium tuberculosis*
NSAID	non-steroidal anti-inflammatory drug
PBTZ	piperazine-containing BTZ
PGE2	prostaglandin E2
RNS	reactive nitrogen species
ROS	reactive oxygen species
TA	toxin–antitoxin
TB	tuberculosis
TDR	total drug resistant
TDM	trehalose dimycolate
TMM	trehalose monomycolate
TNF	tumor necrosis factor
VEGF	vascular endothelial growth factor
WHO	World Health Organization
XDR	Extensively drug resistant
Zmp1	Zn^2+^ metalloprotease
